# Analysis of Blood Donor Deferral Pattern at a Tertiary Care Hospital in Chennai: A Cross-Sectional Retrospective Study

**DOI:** 10.7759/cureus.67541

**Published:** 2024-08-22

**Authors:** Soundharya V, Arthi R, Hari Haran A, Suresh Kumar I, Sahayaraj James

**Affiliations:** 1 Transfusion Medicine, Saveetha Medical College and Hospitals, Saveetha Institute of Medical and Technical Sciences, Saveetha University, Chennai, IND

**Keywords:** non-remunerated, voluntary, transfusion transmissible infections, deferral, donor, blood

## Abstract

Background

Blood is essential for saving lives, particularly in emergencies. However, many patients, especially in developing countries face delays in accessing safe blood due to inadequate infrastructure, insufficient blood banks, poorly equipped laboratories, unreliable transportation systems, low donation rates driven by cultural beliefs, fear, and misconceptions, poor blood donor recruitment and retention, economic constraints, and a general lack of awareness and education about blood donation. Ensuring safe transfusions requires advanced technology and promoting healthy, voluntary donations. Donor selection is crucial for safety, preventing adverse reactions through proper criteria and infection screenings. Donor deferrals can discourage donors and hinder recruitment, so identifying and addressing deferral causes is vital. Blood centers must balance quality and quantity by using thorough donor assessments. Efforts should focus on both recruiting new donors and retaining deferred ones to ensure a stable blood supply.

Aim

The aim of the study is to evaluate and analyze the patterns and causes of blood donor deferrals in a tertiary care hospital. The objectives are to determine the incidence and reasons for blood donor deferrals.

Materials and methods

A cross-sectional retrospective study was conducted for 36 months from May 2021 to May 2024. A simple random sampling method was used to select the blood donors who reported for donation. Data was obtained from records maintained by the blood center. Descriptive statistics were utilized to summarize the demographics of the blood donors, including deferral rates among males and females, and the frequency of temporary and permanent deferrals. A Chi-square test was done to find the association between gender and deferral rates in blood donation. This analysis aimed to explore gender variations and underlying health status differences between male and female donors, as these can influence deferral rates.

Results

From May 2021 to May 2024, 17,082 people registered to donate blood at the Blood Centre, Department of Transfusion Medicine. Out of these, 1,000 donors, or 5.85%, were deferred. The majority of donors were males 16,638, with only 444 females. Most deferrals (76.4%) were temporary, often due to low hemoglobin levels or recent alcohol intake. Permanent deferrals (23.6%) were usually due to uncontrolled hypertension and diabetes. A significant association was found between gender and type of deferral among participants (p < 0.05).

Conclusion

This cross-sectional retrospective study on blood donor deferral patterns in a tertiary care hospital highlights key reasons such as low hemoglobin, recent alcohol intake, hypertension, and diabetes. To improve donor eligibility and retention, targeted strategies including enhanced education and community engagement are essential. These efforts will strengthen blood transfusion services and support critical healthcare needs effectively.

## Introduction

Blood is a lifesaver for critically ill patients, with blood transfusions saving numerous lives worldwide annually. Blood is incredibly vital for human health, especially in emergency conditions where it plays a crucial supportive role [1]. Despite its universal necessity, many patients lack timely access to safe blood, leading to a significant disparity in blood and blood product availability between developed and developing countries [2,3]. Ensuring safe transfusion is critical, requiring the application of science and technology in blood processing and testing, alongside social campaigns to promote blood donation by healthy volunteers who pose minimal infection risk to recipients [2].

Donor selection criteria are essential in blood donation assessment, prioritizing both recipient and donor safety. This process evaluates an individual's suitability to donate blood based on specific standards. Proper donor selection can prevent donor reactions and transfusion-related complications. The safety and availability of blood products hinge on the recruitment and selection of voluntary, non-compensated blood donors. Despite rigorous screening for infectious diseases, donor deferrals can lead to the loss of valuable blood and components needed for transfusion. Understanding the causes and frequency of deferrals is crucial for mitigating this issue [4].

Statistics from the National AIDS Control Organization (NACO) indicate that India donates approximately 7.4 million units of blood annually, falling short of the required 10 million units [5]. The World Health Organization (WHO) reports that over 81 million units of blood are collected globally each year, with only 39% from low-income countries, which represent 82% of the world's population [6]. Ensuring the availability of safe blood requires establishing donor deferral requirements and conducting rigorous screening for potential transfusion-transmissible infections (TTI) [7].

A study done in India has identified various common reasons for blood donor deferral, highlighting different demographic characteristics across the country [1]. Donor deferral can be a challenging and unpleasant experience for both the donor and the blood facility. These deferrals hamper efforts to recruit new donors and often result in potential donors feeling negative about themselves and the donation process [1]. However, the enforcement of deferral criteria significantly influences the quality of blood supplied to a large population. Each blood center must carefully balance maintaining acceptable quality and achieving desired quantity [8].

A fundamental tool for donor selection is the "donor questionnaire," which assesses the donor's history and health to determine infection risk and eligibility. A confidential interview between the donor and a staff member ensures thorough evaluation [2]. However, NACO and State Blood Transfusion Councils (SBTCs) do not regularly oversee donor deferrals, focusing instead on the quantity of supply and deferrals based on viral indicators found in donated units. Efforts often prioritize recruiting new donors over re-engaging individuals who were previously deferred [1].

The primary objective of this study is to evaluate and analyze the patterns and causes of blood donor deferrals in a tertiary care hospital, focusing on the incidence and specific reasons for these deferrals. The study aims to systematically investigate all critical factors collected during donor selection, including donor demographics, medical history, physical examination findings, hemoglobin levels, vital signs, and any risk behaviors. By aligning with the donor selection criteria established by the NACO guidelines, the WHO, and the Drugs and Cosmetic Act amendment of 2020, the study seeks to identify the most common causes of donor deferrals. Understanding these factors is crucial for refining the blood donation process, reducing deferrals, and ensuring a consistent and safe blood supply. Ultimately, the study intends to enhance donor retention and re-engagement, contributing to the availability and safety of blood products for transfusion.

## Materials and methods

Study design and setting

This study was a cross-sectional retrospective analysis focusing on voluntary non-remunerated blood donors who reported for blood donation. It was conducted at the Blood Center, Department of Transfusion Medicine, in a tertiary care hospital in Chennai, Tamil Nadu. Ethical approval from the Institutional Review Board was obtained before the commencement of the study. The study involved retrieving data recorded in the donor deferral register of our blood center. The donor deferral register provided comprehensive information about each deferred donor, including reasons for deferral and other relevant details. This register served as the primary data source for analyzing the patterns and causes of blood donor deferrals in the study.

The study focuses on the proper selection of voluntary, non-remunerated blood donors at a tertiary care hospital and aims to evaluate and analyze the patterns and causes of blood donor deferrals. It adheres to NACO guidelines, WHO standards, and the 2020 Drugs and Cosmetic Act amendment. The evaluation process includes assessing the incidence and reasons for deferrals by examining donor demographics, medical history, physical examination findings, hemoglobin levels, vital signs, and risk behaviors. This comprehensive approach is designed to identify the most common causes of donor deferrals.

Study participants

Inclusion Criteria

All voluntary, non-remunerated blood donors who came forward for donation were included, and all donors who participated in the voluntary blood donation camps. Donors who met the Blood Centre's health and eligibility conditions, including acceptable hemoglobin levels between 12.5-16.5 g/dl and normal blood pressure, heart rate, temperature, and saturation, were included. Donors who provided informed consent to use their data for research purposes were included. Individuals within the age range specified by the Blood Centre’s guidelines (typically 18-60 years) were included.

Exclusion Criteria

Donors who did not meet the health criteria for donation, including: a) Hemoglobin levels not within acceptable range; b) Abnormal blood pressure (systolic outside 100-140 mmHg or diastolic outside 60-90 mmHg); c) Disqualifying health conditions such as anemia, cardiovascular diseases, diabetes, recent surgery, or blood disorders were excluded. Donors with incomplete or missing data in their medical records and donors who failed to comply with standard donation procedures or were deemed non-compliant with Blood Centre protocols were excluded.

Study size and sampling method

The study included 17,082 registered blood donors over the 36-month period. A simple random sampling method was done to select blood donors, ensuring a representative sample of the donor population.

Data collection

Data were collected over 36 months, from May 2021 to May 2024. Information was obtained from records meticulously maintained by the Blood Centre, including the voluntary blood donor registry. Each donor's medical history was reviewed, and a brief physical examination was conducted by a doctor, focusing on key health indicators such as hemoglobin levels, blood pressure, temperature, pulse rate, and regularity. Data were systematically organized in Excel (Microsoft, Redmond, WA, USA). The study adhered to stringent criteria set by the NACO, WHO, the Drugs and Cosmetics Act amendment of 2020, and the Directorate General of Health Services (DGHS) 2022, ensuring the reliability of the collected data [9].

Primary variables

Donor demographics (age, gender), health indicators (hemoglobin levels, blood pressure, temperature, pulse rate), and reasons for deferral (temporary or permanent) were studied. Deferred donors were classified by gender, age group, and deferral nature (temporary or permanent) to identify patterns and trends, understand health implications, improve blood donation policies, and enhance donor recruitment.

Statistical methods

Descriptive statistics were employed to summarize donor demographics, deferral rates among males and females, and to assess the frequency of temporary and permanent deferrals. Additionally, a Chi-square test was conducted to examine the association between gender and deferral rates.

## Results

**Figure 1:**
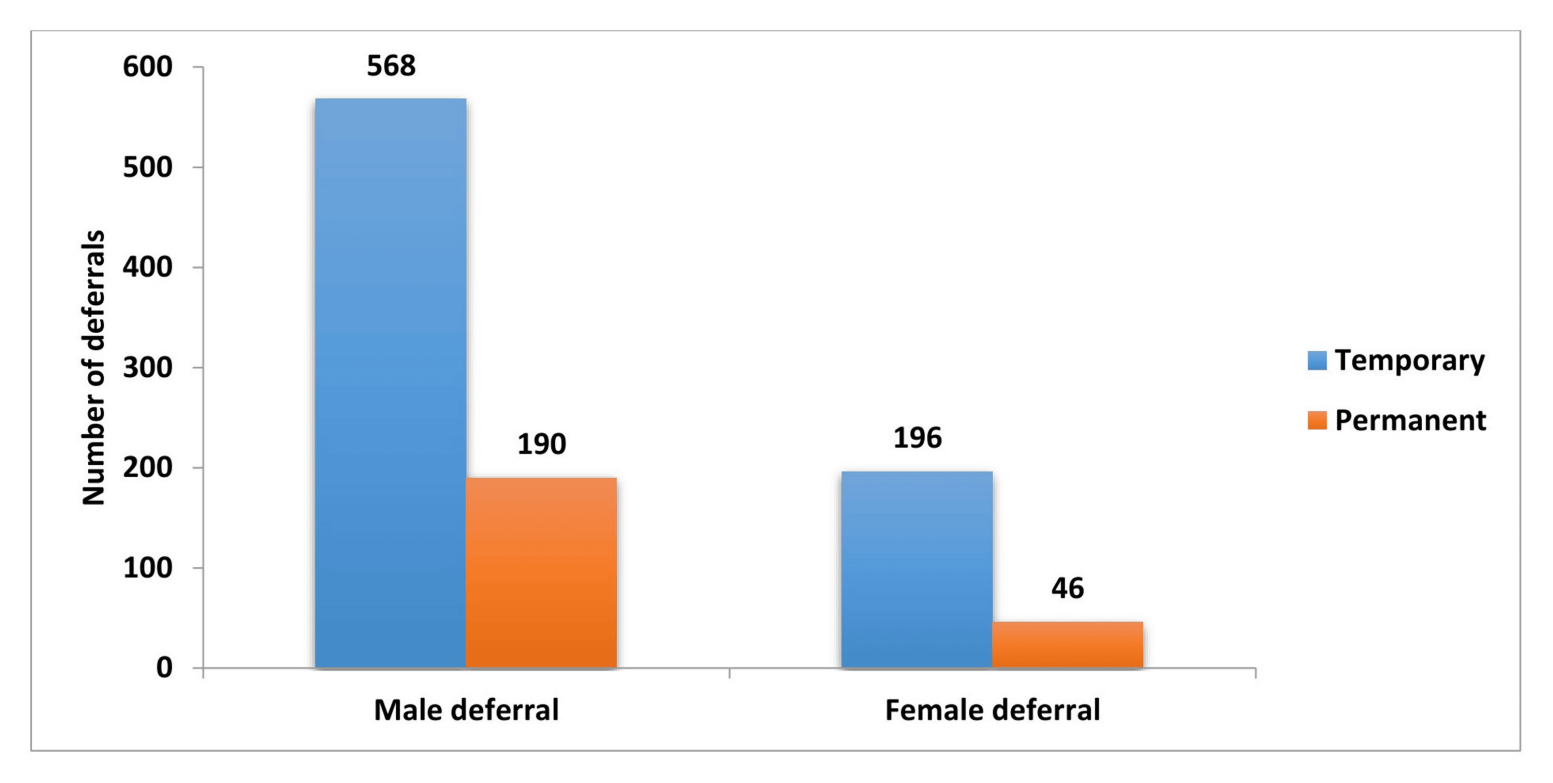
Number of temporary and permanent deferrals among male and female donors

A total of 17,082 participants registered to donate blood at the Blood Center, Department of Transfusion Medicine, between May 2021 and May 2024. Of these, 16,638 were males (97.4%) and 444 were females (2.6%). The overall deferral rate for all donors was found to be 5.8% (Table 1, Figure 1). A closer examination of deferral rates revealed a significant gender disparity: 4.5% of the registered males were deferred, while a much higher 54.5% of the registered females were deferred. Despite their lower deferral rate, males accounted for 75.8% of the total deferrals due to their larger representation among donors, whereas females, though a smaller portion of the donor pool, represented 24.2% of the total deferrals.

**Table 1:** Demographics of donors and deferral rate

Gender	Registered for donation (N)	No. of deferrals (N)	% of deferrals
Male	16638	758	4.5%
Female	444	242	54.5%
Total	17082	1000	5.8%

Table 1 shows that out of 17,082 registered blood donors, males had a deferral rate of 4.5%, while females had a much higher deferral rate of 54.5%. Despite their lower deferral rate, males accounted for the majority of deferrals due to their larger representation among donors.

**Figure 2:**
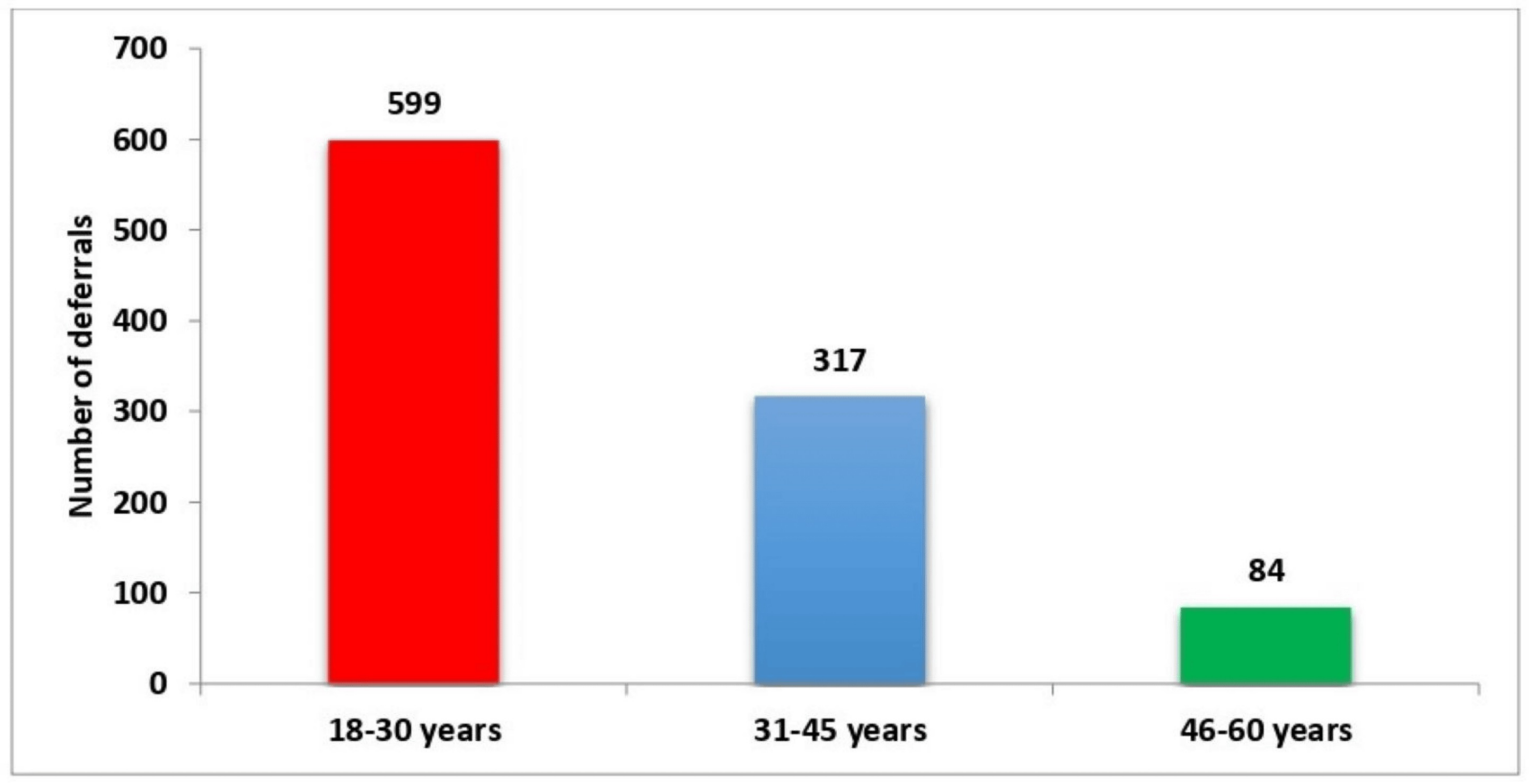
Distribution of deferred donors by age

The age distribution of deferred donors showed that the majority were younger, with 59.9% (599 individuals) falling within the 18- to 30-year age group. Another 31.7% (317 individuals) were aged between 31 and 45 years, and 8.4% (84 individuals) were aged between 46 and 60 years (Table 2, Figure 2).

**Table 2:** Age distribution of the deferred donors

Age group of deferred donors	Number of donors deferred (N)	% of donors deferred
18-30 years	599	59.9
31-45 years	317	31.7
46-60 years	84	8.4
Total	1000	100

Table 2 shows that most deferred donors were aged 18-30 years (59.9%), followed by those aged 31-45 years (31.7%). Donors aged 46-60 years accounted for 8.4% of the deferrals.

Donor deferrals were categorized into two types: temporary and permanent. Temporary deferrals were more common, constituting 76.4% (764 individuals) of all deferrals, compared to 23.6% (236 individuals) for permanent deferrals. Among the temporarily deferred, 74.34% (568 individuals) were men and 25.66% (196 individuals) were women (Table 3). In the permanent deferral category, 10.95% were males and 2.80% were females.

**Table 3:** Frequency of temporary and permanent deferrals

Type of deferral	Number of deferrals	Male deferrals N (%)	Female deferrals N (%)	% of deferral
Temporary	764	568 (74.93)	196 (80.99)	76.4
Permanent	236	190 (25.06)	46 (19.01)	23.6
Total deferral	1000	758	242	100

Table 3 reveals that 76.4% of deferrals were temporary, with 74.93% being males and 80.99% being females. Permanent deferrals made up 23.6%, with 25.06% being males and 19.01% being females.

Table 4 shows a significant association between gender and the type of deferral, with a chi-square value of 3.7333 and a p-value less than 0.05, indicating a statistically significant relationship. The data shows that 74.93% of males and 80.99% of females were temporarily deferred, while 25.06% of males and 19.01% of females were permanently deferred. This indicates that females are more likely to experience temporary deferrals, suggesting that they may encounter health issues that are generally short-term and resolvable. Conversely, males are more frequently subject to permanent deferrals, which typically indicate more serious or chronic health conditions. These findings suggest that gender influences the likelihood of deferral type, with females more often facing temporary deferrals and males more frequently experiencing permanent deferrals.

**Table 4:** Association of gender with temporary and permanent deferrals *p-value <0.05- significant (Pearson’s Chi-Square test)

Type of deferral	Male deferrals N (%)	Female deferrals N (%)	Chi square	p-value
Temporary	568 (74.93)	196 (80.99)	3.7333	< 0.05*
Permanent	190 (25.06)	46 (19.01)		

The most common reason for temporary deferrals was low hemoglobin levels, accounting for 73.70% (563 cases) of all temporary deferrals. The next most frequent cause was alcohol intake within 24 hours prior to donation, responsible for 11.91% (91 cases) of temporary deferrals. The least frequent reasons included a history of surgery within 12 months prior to donation and, for females, deferral due to menstruation, each accounting for 0.53% (four cases) and 0.30% (four cases), respectively (Table 5).

**Table 5:** Factors leading to temporary deferral

Causes for deferral	No. of deferrals	% of temporary deferrals	% of total deferral
Low Hemoglobin	563	73.70	56.3
Alcohol intake	91	11.91	9.1
Low weight	46	6.02	4.6
Tattoo within 1 year	21	2.74	2.1
Cold / Fever	13	1.70	1.3
Medications- Antibiotics	10	1.30	1.0
Recent donation within 3 months	7	0.92	0.7
Vaccination history	5	0.65	0.5
Inadequate sleep previous night	4	0.53	0.4
Menstruation	4	0.53	0.4
Total	764	100	76.4

Table 5 shows that low hemoglobin was the leading cause of temporary deferrals (73.70%), followed by alcohol intake (11.91%). Other reasons included low weight and recent tattoos. Temporary deferrals accounted for 76.4% of all deferrals.

Table 6 reveals that uncontrolled hypertension is the most prevalent cause of permanent blood donor deferrals, accounting for 53.39% of permanent deferrals and contributing to 12.6% of all deferrals. Uncontrolled diabetes mellitus is the second most frequent cause, representing 26.28% of permanent deferrals and 6.2% of total deferrals. The least common cause of permanent deferral is epilepsy, which accounts for 3.38% of permanent deferrals.

**Table 6:** Factors leading to permanent deferral COPD: Chronic obstructive pulmonary disease

Causes for deferral	Number of donors	% of permanent deferrals	% of total deferral
Uncontrolled Hypertension	126	53.39	12.6
Uncontrolled diabetes mellitus	62	26.28	6.2
COPD/Asthma	17	7.21	1.7
Cardiac Diseases	12	5.08	1.2
Age >60yrs	11	4.66	1.1
Epilepsy	8	3.38	0.8
TOTAL	236	100	23.6

Overall, permanent deferrals constituted 23.6% of all deferrals, with the remaining 76.4% being temporary. The data highlights that specific health conditions, such as hypertension and diabetes, play a significant role in the likelihood of permanent deferral, whereas other conditions like epilepsy are less common.

These findings indicate that gender, age, and specific health conditions play a significant role in determining blood donor deferral. The stringent donor deferral criteria, as outlined by DGHS, NACO, and WHO guidelines, underscore specific reasons for deferral within the population. This emphasizes the need for targeted strategies to reduce the deferral rate. By addressing the prevalent health issues that cause deferrals, we can work toward improving the overall donation rate, thereby ensuring a more robust and reliable blood supply. This approach not only enhances donor retention but also strengthens the effectiveness of transfusion services, ultimately benefiting patient care.

## Discussion

The primary role of the Blood Transfusion Service (BTS) is to ensure a safe, adequate, and timely supply of blood and blood products, which is crucial for both donor and recipient safety. Achieving this goal requires a rigorous donor screening process to avoid jeopardizing the health of donors and to maintain the integrity of the blood supply. A well-structured and secure screening protocol is essential for identifying potential health risks and ensuring that only suitable candidates are accepted for donation. Conversely, inadequate management and lax adherence to deferral policies can lead to the loss of valuable donors and compromise the operational efficiency of blood banks, which are already struggling with a chronic shortage of blood units. The lack of overview, knowledge, and statistics about blood donation in society is the cause of this entire scenario [10].

The current study's deferral rate of 5.85% aligns closely with findings from previous research, including those by John et al. [11] (5.12%), Unnikrishnan et al. [12] (5.20%), Sundar et al. [13] (6%), and Rabeya et al. [14] (5.6%). Male donors experienced a deferral rate of 75.8%, while female donors had a deferral rate of 24.2%. These findings are consistent with the research conducted by Sundar et al. [13], Gaajreetal [15], Agnihotri et al. [16] and Taneja et al. [17]. The lower participation and deferral rates among female donors are largely attributed to the misconception that women are less suitable for blood donation due to a higher prevalence of anemia, as well as fear and lack of awareness regarding the donation process.

The present study reveals a higher rate of deferrals among individuals aged 18-30 years, making up 59.9% of all deferrals. This finding is consistent with data reported by Shah et al. [18] and Sundar et al. [13]. While young adults tend to be the most eligible and readily available donors compared to older individuals, they also exhibit a higher deferral rate within this demographic group.

The temporary deferral rate, at 76.4%, surpasses the permanent deferral rate of 23.6%, aligning with earlier research by Kulkarni [19], Custer et al. [20], John et al. [11], Rehman et al. [21], Chauhan et al. [22], Kujur et al. [23], and Malini et al. [24]. In the current study, anemia emerged as the leading cause of temporary deferral, accounting for 73.70% of cases, followed by alcohol intake at 11.91%. Numerous studies have consistently identified anemia as the predominant reason for temporary deferral. Table 7 provides a comparative analysis of the various causes of deferral across multiple studies.

**Table 7:** Temporary deferral comparison

Causes for temporary deferral	Present study (%)	Chitra Chauhan et al ^[22]^ (%)	K Faheem et al ^[10]^ (%)	Pratima Kujur et al ^[23]^ (%)	Bhimani et al ^[1]^ (%)	Padma Malini et al^ [24]^ (%)	S K Chavan et al ^[3]^ (%)
Low hemoglobin	73.70	42.26	7.72	16.7	53.59	19.90	77.70
Alcohol intake	11.91	17.68	26.26	32.08	4.57	15.93	2.09
Low weight	6.02	4.49	24.29	5.53	5.23	15.93	1.85
Tattoo within 1 year	2.74		5.61	1.14	5.23	5.97	-
Cold / fever	1.70	1.66	-	1.6	1.96	-	-
Medications like antibiotics	1.30	5.11	-	7.32	3.27	-	9.29
Recent donation within 3 months	0.92	5.80	1.96	6.67	2.62	-	0.46
Vaccination	0.65	0.55	3.23	1.30	-	0.39	0.92
Inadequate sleep	0.53	0.14	9.97	0.48	-	15.93	-
Menstruation	0.53	-	1.68	2.28	6.53	3.98	0.11

In the current study, hypertension emerged as the leading cause of permanent deferral, accounting for 53.39% of cases, followed by uncontrolled diabetes mellitus at 26.28%. These findings are consistent with those reported in previous studies conducted by Kujur et al. [23], Bhimani et al. [1], Faheem et al. [10], Chavan et al. [3], Chauhan et al. [22], and Sabari Priya et al. [4]. Table 8 tabulates the comparison of several studies of permanent deferral and its causes. 

**Table 8:** Permanent deferral comparison COPD: Chronic obstructive pulmonary disease

Causes for permanent deferral	Present study (%)	Pratima Kujur et al ^[23]^ (%)	Bhimani et al ^[1] ^(%)	K Faheem et al ^[10]^ (%)	S K Chavan et al ^[3] ^(%)	Chitra Chauhan et al ^[22]^ (%)	Sabari Priya et al ^[4]^ (%)
Uncontrolled hypertension	53.39	88.05	49.35	15.66	16.99	1.93	27.63
Uncontrolled diabetes mellitus	26.28	5.08	24.70	2.68	1.18	0.35	7.21
COPD/asthma	7.21	-	10.38	4.00	-	0.14	
Cardiac diseases	5.08	-	6.48	-	2.37	1.93	
Age >60yrs	4.66	-	-	-	-	0.35	
Epilepsy	3.38	1.69	3.90	2.00	-	-	0.90

Our findings align with previous research, indicating a significant association between gender, age, and the type of deferral. The study observed higher deferral rates among males and younger individuals, which underscores the need for targeted strategies to address specific demographic challenges. 

Regular monitoring and stringent health checks during the donation process also contribute to maintaining the overall safety of blood transfusions [14]. Assessments of social behaviors and lifestyle factors are equally important. This includes evaluating factors such as alcohol consumption, drug use, and travel history, which can impact the donor's eligibility and the safety of the blood supply. By understanding these social and behavioral aspects, blood banks can better manage deferral risks and address issues that may contribute to higher deferral rates [19].

To ensure a safe and adequate supply of blood for recipients, it is essential to implement a comprehensive strategy involving detailed donor histories, thorough physical and clinical examinations, and evaluations of social behaviors. A detailed donor history provides critical insights into potential health risks and past medical conditions that could affect the donor’s eligibility and the safety of the donated blood. This history helps identify any pre-existing conditions or lifestyle factors that might disqualify a donor or increase the risk of complications, thereby enabling the BTS to make informed decisions regarding donor acceptance [10]. The integration of these practices into blood donation strategies will aid in overcoming the challenges associated with donor deferral and improve overall operational efficiency. 

Limitations of the study

This study has certain limitations. As a retrospective study, it relies on previously collected data, which may be incomplete, affecting accuracy and reliability. Conducted at a single tertiary care hospital in Chennai, its results may not be generalizable to other regions. Therefore, the results might not be applicable to other populations or settings, limiting the broader applicability of the conclusions drawn from this study. The lower number of female donors compared to male donors might not fully capture deferral reasons specific to females, potentially skewing findings. The retrospective nature of the study lacks follow-up data on deferred donors to determine if they eventually became eligible to donate. Covering the period from May 2021 to May 2024, the findings may not be applicable to periods outside the study duration. Additionally, the study does not explore sociocultural factors that might influence donor deferrals. Factors such as religious beliefs, cultural taboos, or social norms can significantly impact donor behavior and deferral rates, especially in diverse populations.

## Conclusions

Despite a relatively low donor deferral rate of 5.85% in the present study, various reasons and social taboos seem to have intertwined and affected the entire scenario of blood donation. Social taboos and cultural beliefs significantly impact blood donation rates, often deterring individuals from participating. Deep-seated cultural superstitions and religious doctrines can create fears and misconceptions about the safety and implications of donating blood, such as concerns about weakness or health risks. Stigma and misinformation about the donation process further contribute to reluctance, with some individuals fearing pain or potential exposure to infections. Social and gender norms may also play a role, with misconceptions about who is suitable for donation, and privacy concerns can exacerbate reluctance. Additionally, a lack of awareness about the benefits and safety of blood donation can perpetuate these taboos. Addressing these barriers through targeted education, community engagement, and efforts to dispel myths is crucial for increasing donation rates and ensuring a steady and reliable blood supply.

Anemia and alcohol consumption were the leading causes of donor deferral, reflecting the societal norms and overall health status of our community. As a result, individuals must realize the value of blood donation, and youth should take active actions to support, mobilize, and promote this practice. Additionally, to become eligible donors, they must avoid certain detrimental social behaviors within a suitable time frame. Stringent blood donor selection according to the standard guidelines has to be followed for a safe blood supply and also to create awareness among the donors to improvise their health status. The BTS and blood centers ought to implement significant steps to promote blood donation while simultaneously addressing the community's social and health needs. This approach will help avoid the unwarranted deferral of potential and healthy blood donors. Community awareness, public education campaigns, and gatherings can contribute to creating a more informed society with a healthier donor population, ensuring optimal blood donation services.
